# Conditions for Knowledge and Application of Vegetarian/Vegan Diets Among Secondary School Students: A Cross-Sectional Study

**DOI:** 10.3390/nu18081210

**Published:** 2026-04-11

**Authors:** Oliwia Kurzawska, Ewa Raczkowska

**Affiliations:** Department of Human Nutrition, Faculty of Biotechnology and Food Science, Wrocław University of Environmental and Life Sciences, 37 Chełmońskiego Street, 51-630 Wrocław, Poland; 122374@student.upwr.edu.pl

**Keywords:** vegetarian diet, vegan diet, plant-based diet, nutritional knowledge, BMI, adolescents

## Abstract

**Background/Objectives**: Knowledge of plant-based diets is gaining increasing significance in adolescents due to the growing popularity of vegetarian and vegan dietary patterns. To date, there has been limited research examining the level of awareness and understanding of these diets among secondary school students, as well as the factors influencing their knowledge. The aim of the study was to determine the prevalence of plant-based diets and to assess knowledge regarding these dietary patterns among high school students, as well as to identify factors associated with both diet adherence and achieving sufficient nutritional knowledge. **Methods**: A cross-sectional study was conducted among 341 high school students. Data were collected using a self-administered paper questionnaire that included demographic information, self-reported body weight and height, adherence to plant-based diets, and knowledge of vegetarian and vegan nutrition. Nutritional knowledge was assessed using a structured 19-item questionnaire (25 scorable items) and verified for reliability (test–retest, Krippendorff’s alpha = 0.88). Based on a 25-point scale, a score of >60% (16–25 points) was categorized as ‘sufficient’ knowledge. Statistical analyses included the chi-square test, Mann–Whitney and Kruskal–Wallis non-parametric tests, and multivariable logistic regression to estimate adjusted odds ratios (aOR) for factors associated with sufficient knowledge. **Results**: The prevalence of plant-based diets in the study group was 16.1% (*n* = 55), with a significantly higher frequency observed among female students and those with sufficient nutritional knowledge. The majority of students (81.2%) achieved sufficient knowledge. Higher scores were observed among female students, those in higher grade levels, and those individuals adhering to plant-based diets (*p* < 0.05). Multivariate regression analysis indicated that male sex (aOR = 0.38 compared to females), higher grade level (aOR = 3.66 for grade 3 vs. grade 1; aOR = 3.62 for grade 4 vs. grade 1), residence in a rural area (aOR = 0.50), and non-adherence to a plant-based diet (aOR = 0.32) were independently associated with sufficient knowledge. **Conclusions**: The majority of high school students demonstrate sufficient knowledge regarding plant-based diets, with significant variations associated with sex, grade level, place of residence, and experience with plant-based diets. These findings underscore the need for targeted educational interventions, particularly among male students, those in lower grade levels, and individuals residing in rural areas.

## 1. Introduction

Currently, an increasingly pronounced trend toward plant-based diets is being observed both globally and in Poland. Interest in vegetarianism and veganism is reflected, among other indicators, in the rising frequency of internet searches related to these topics. Changes in dietary attitudes are accompanied by intensified scientific debate concerning the benefits and limitations of plant-based diets, particularly when implemented in populations with specific nutritional requirements, such as children and adolescents [1].

Vegetarianism is defined as a dietary pattern that excludes meat, fish, and seafood, while veganism eliminates all animal-derived products, including dairy, eggs, and honey [2,3]. Less restrictive forms of plant-based diets also exist, differing in their degree of exclusion and potential risk of nutrient deficiencies (lacto-vegetarianism, ovo-vegetarianism, pescovegetarianism) [4].

Global and Polish data indicate that the proportion of individuals avoiding animal products is increasing, particularly among youth [5,6,7,8]. In Poland, up to 21.7% of individuals aged 18–24 reported not consuming meat in the month preceding a recent survey, representing the highest proportion across all age groups [9]. While recent studies have examined the health status of younger Polish children (aged 5–10) following vegetarian and vegan diets [10], research focusing specifically on secondary school students remains scarce.

The safety of plant-based diets in pediatric populations remains a subject of scientific and public debate. Proper dietary balance is crucial during early life for optimal growth and skeletal health. The Committee on Human Nutrition Science of the Polish Academy of Sciences (PAN) suggests that while some plant-based variants may provide essential nutrients, restrictive veganism is generally not recommended for older children and adolescents [11]. Conversely, the ESPGHAN Committee on Nutrition considers these diets acceptable if strict medical and dietary guidelines are followed [12]. However, the risk of non-compliance and subsequent deficiencies—particularly of vitamin B_12_, iron, calcium, iodine, and omega-3 fatty acids—is substantial [13,14].

While nutritional risks for young children are well-documented, secondary school students represent a critical group characterized by increasing dietary autonomy, the influence of social media, and body image pressures [15]. Despite this, there is a lack of research evaluating whether their dietary choices, often driven by ethics, environment, or health, are supported by adequate nutritional knowledge. Given the potential health risks and the rising popularity of these dietary patterns, this study aimed to determine the prevalence of plant-based diets and to assess the level of knowledge regarding vegetarian and vegan nutrition among high school students. Furthermore, we sought to identify factors associated with both diet adherence and the attainment of sufficient nutritional knowledge, including sex, grade level, place of residence, and BMI. In the present study, the term plant-based diet refers to dietary patterns encompassing both vegetarian and vegan diets.

## 2. Materials and Methods

### 2.1. Study Design and Sample

The study had a cross-sectional design and was conducted among students attending a single general secondary school in Wrocław, Poland. A convenience sampling method was employed, selecting an institution where the researchers were granted permission to conduct the study. This school was specifically chosen due to its diverse educational structure, comprising various profiles such as humanities, natural sciences (biology–chemistry-oriented), psychology, and an academic track focusing on mathematics, English, and a choice of science subjects (biology, physics, or geography). A total of 341 students participated. Preliminary statistical analysis showed no significant differences in nutritional knowledge or adherence to plant-based diets between the different educational profiles (*p* > 0.05); therefore, the data were analyzed as a single cohort to maintain statistical power. The study aimed to capture both the theoretical understanding of plant-based nutrition and the practical application (adherence) of these diets in the adolescent population.

For the purpose of statistical analysis, participants were divided into two main groups: those following a traditional omnivorous diet and those categorized as ‘ever-adhered’ to a plant-based diet. The ‘ever-adhered’ group included both current practitioners and those who had followed a vegetarian or vegan diet in the past. This approach was adopted to capture the nutritional knowledge and experiences of all students who had made a conscious decision to limit or exclude animal products, regardless of their current dietary status. Participants were categorized based on their self-declaration in the questionnaire. For those reporting adherence, additional data were collected regarding the duration of the diet (ranging from <6 months to >2 years), the primary motivations for adopting a plant-based pattern (e.g., ethical, health, or environmental concerns), and professional dietary supervision.

The questionnaire was administered to all students in the selected classes during their scheduled lessons, following the teacher’s permission. Participation was voluntary, and students who did not wish to take part were not included. Data collection took place from April to November 2025.

Assuming a significance level of α = 0.05 and a statistical power of 80%, the minimum required sample size for the planned analyses was estimated at approximately 220 participants. The final sample size of 341 individuals exceeded this threshold, indicating sufficient statistical power to detect moderate effect sizes.

The inclusion criteria were: status as a secondary school student, age corresponding to high school attendance, voluntary consent to participate in the study, and completeness of the questionnaire responses. The exclusion criteria comprised lack of consent to participate and incomplete completion of the questionnaire. The study was conducted in accordance with the principles of the Declaration of Helsinki [16]. Participants were informed about the purpose of the study, its anonymous nature, and their right to withdraw at any stage. Written informed consent was obtained from all participants. In the case of participants under 18 years of age, written consent was also obtained from their parents or legal guardians prior to participation in the study. The study protocol was approved by the Research Ethics Committee of the Wrocław University of Environmental and Life Sciences (No. 14/2023, dated 29 June 2023).

### 2.2. Research Tools

Data were collected using an original, structured questionnaire developed by the authors, which was distributed to participants for self-completion in paper form during school classes.

The instrument was divided into three main sections:

- Socio-demographic and personal details: This section was adapted from the validated KomPAN^®^ questionnaire (Committee on Human Nutrition Science of the Polish Academy of Sciences) [17]. It included questions regarding among others sex, age, place of residence.
- Nutritional knowledge test: This component consisted of 19 questions, resulting in 25 scorable items (17 single-choice and 2 multiple-response questions). The content was developed based on an extensive literature review [18,19,20] to cover both general nutrition and specific aspects of plant-based diets. Each correct item was awarded one point, with a maximum total score of 25.
- Plant-based diet section: A targeted section for respondents declaring adherence to plant-based diets to further explore their dietary motivations and practices.

The reliability of the instrument was verified using the test–retest method on a group of 20 students. The stability of the responses was assessed using Krippendorff’s alpha coefficient, yielding a value of 0.88. This result indicates very good test–retest reliability and high stability of the research tool over time. This coefficient was selected for its robustness in handling various scales of measurement used in the questionnaire. The complete questionnaire, including the socio-demographic section, the nutritional knowledge test, and the section dedicated to plant-based diet practitioners, is provided in the Supplementary Materials (S1).

### 2.3. Assessment of the Nutritional Status of Students

The assessment of students’ nutritional status was conducted using the body mass index (BMI), calculated on the basis of self-reported anthropometric data obtained from the questionnaire (body weight [kg]/height [m]^2^). For respondents under 18 years of age, nutritional status was classified according to the WHO BMI-for-age growth reference (z-scores), distinguishing the following categories: severe thinness (<−3 SD), thinness (<−2 SD), normal body weight, overweight (>+1 SD), and obesity (>+2 SD). For adult respondents, nutritional status was classified based on BMI values as follows: <18.5 kg/m^2^—underweight; 18.50–24.99 kg/m^2^—normal body weight; 25.00–29.99 kg/m^2^—overweight; ≥30.0 kg/m^2^—obesity [21]. For the purposes of statistical analysis, detailed categories of nutritional status were aggregated into three groups: below normal (severe thinness and underweight), normal (normal body weight), and above normal (overweight and obesity).

### 2.4. Assessment of Students’ Nutritional Knowledge

The level of students’ nutritional knowledge was assessed using a structured questionnaire consisting of 19 questions, which translated into a total of 25 scorable items. For 17 single-choice questions, one point was awarded for each correct answer. For two multiple-response questions, respondents received one point for each correctly identified component, resulting in a maximum possible score of 25 points. The questions spanned a range of difficulty levels, from fundamental diet definitions to complex issues concerning nutrient interactions and bioavailability. A threshold of >60% correct responses (16–25 points) was established to categorize knowledge as ‘sufficient.’ This cut-off point was selected based on established pedagogical standards in Poland and previously published nutritional literacy studies [22,23], where it serves as a robust benchmark for distinguishing between insufficient and satisfactory levels of specialized knowledge.

### 2.5. Statistical Analysis

Statistical analyses were performed using Statistica 13.3 software (StatSoft^®^, Tulsa, OK, USA). The normality of variable distributions was assessed using the Shapiro–Wilk test. As the distributions deviated from normality, nonparametric tests were applied for group comparisons: the Mann–Whitney U test for comparisons between two groups and the Kruskal–Wallis test for comparisons among three or more groups. Preliminary analyses, including the Kruskal–Wallis test for nutritional knowledge scores and the chi-square test for the prevalence of plant-based diets, were performed to identify potential differences between the educational tracks. As no statistically significant differences were found (*p* > 0.05), the data from all tracks were pooled into a single cohort to enhance statistical power for the subsequent multivariable logistic regression models. Potential outliers in continuous variables, such as knowledge scores and BMI, were assessed using boxplots and the interquartile range (IQR) method. Values falling below Q1 − 1.5 × IQR or above Q3 + 1.5 × IQR were examined for plausibility, and all verified data points were retained in the analysis. Categorical variables are presented as frequencies (N) and percentages (%). Differences between categorical variables—such as sex, place of residence, grade level, nutritional status, adherence to plant-based diets, and level of nutritional knowledge—were evaluated using the chi-square test.

To assess independent associations between a sufficient knowledge regarding plant-based diets and demographic and anthropometric factors, multivariable logistic regression analysis was performed. The model was built using the ‘enter’ method, where all potential predictors were entered simultaneously to evaluate their combined and individual contributions. The selection of independent variables—including age, sex, BMI, place of residence, grade level, and adherence to plant-based diets—was based on their documented theoretical relevance as potential determinants of nutritional literacy in adolescents [24,25]. This multivariable approach was specifically employed to handle potential confounding; by including all relevant socio-demographic factors in a single model, we were able to estimate adjusted odds ratios (aORs) for each variable while controlling for the influence of others. Multicollinearity between independent variables was rigorously assessed using variance inflation factors (VIF), and no evidence of problematic collinearity was observed. Model fit was confirmed using the Hosmer-Lemeshow goodness-of-fit test (*p* > 0.05). Missing data were minimal and handled using a complete-case analysis approach. Continuous variables, such as BMI, were categorised to facilitate clinical interpretation and align with established classification criteria. A *p*-value of <0.05 was considered statistically significant for all analyses.

## 3. Results

### 3.1. Characteristics of the Study Group

Table 1 presents the socio-demographic characteristics of the studied group of high school students (N = 341). The study population consisted predominantly of females, who accounted for nearly three-quarters of the participants, whereas the proportion of males was considerably lower. For subsequent statistical analyses examining sex-based differences, only participants who declared their sex as female or male were included. Individuals who did not report their sex were considered solely in the descriptive socio-demographic characterization of the study group. The majority of students resided in large urban areas (65.1%), while rural areas and medium-sized cities were less represented (24.6% and 10.3%, respectively). Analysis of body mass index indicated that the vast majority of respondents fell within the normal range (83.3%), whereas the proportions of students classified as underweight or overweight were relatively low. The distribution of students by grade level revealed the highest representation in the second and third grades (approximately 29% each), while first- and fourth-grade students constituted smaller proportions of the study population.

In the study group, 55 students declared that they currently follow or have followed a plant-based diet in the past. The largest percentage of respondents (32.73%) had followed the diet for a period not exceeding 6 months, whilst 27.27% of those questioned declared that they had followed it for over 2 years. Respondents were also asked to indicate the reasons for adopting a plant-based diet. The most frequently reported reason was ethical considerations (72.73%), followed by environmental concerns (29.09%), whilst health reasons were the least frequently cited (14.54%). In addition, the students were asked whether they had consulted a dietitian about their diet. Only 20% of respondents stated that they had sought such a consultation (Table 2).

### 3.2. Level of Nutritional Knowledge About Plant-Based Diets

The mean score on the plant-based diet knowledge test in the study group was 17.9 ± 2.8 points, with a median of 18 points (Q_1_ = 16; Q_3_ = 20) and a range from 9 to 25 points. Analysis of knowledge levels indicated that the majority of students (81.2%) achieved a sufficient knowledge, whereas 18.8% of participants demonstrated an insufficient level (Table 3).

**Figure 1:**
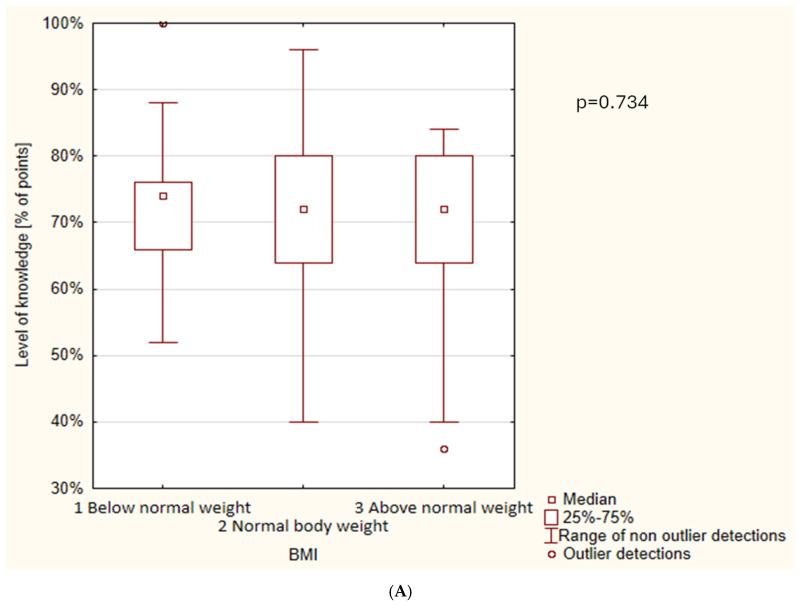

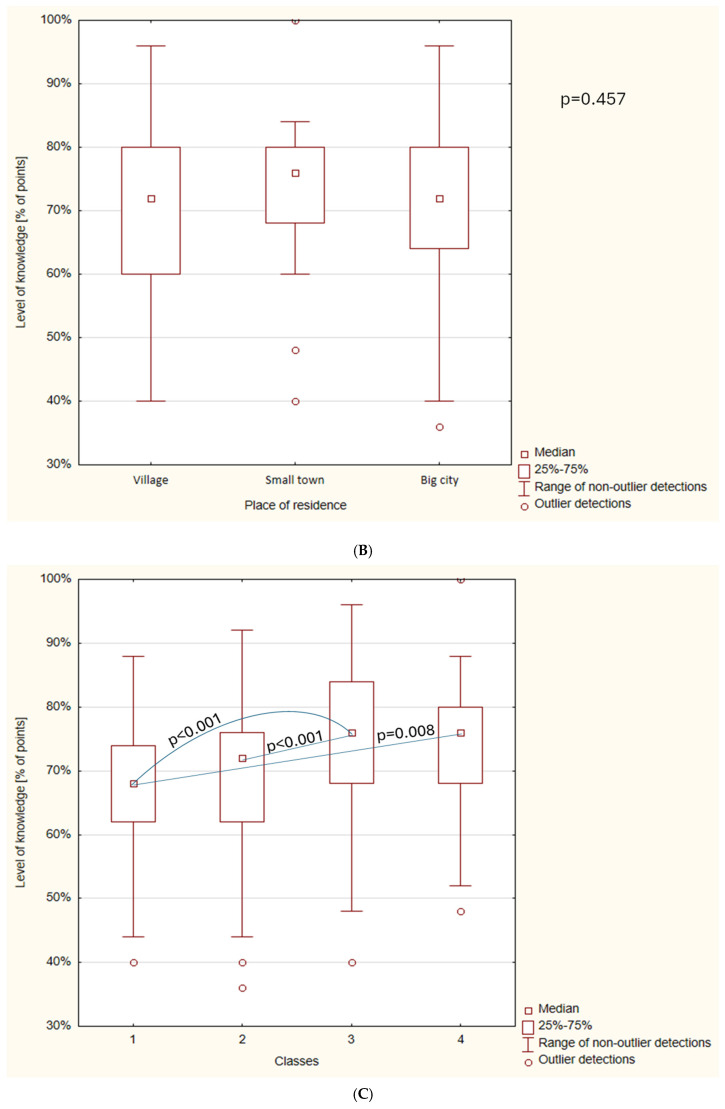

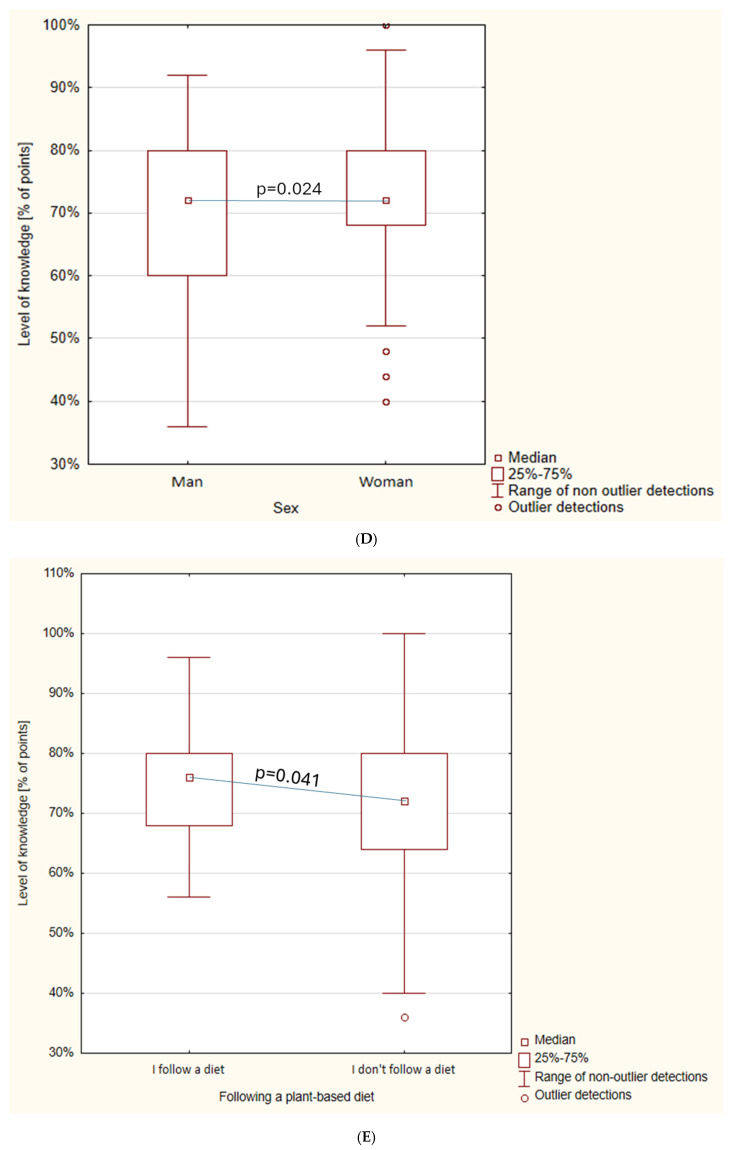
Level of nutritional knowledge depending on: nutritional status (BMI) (**A**); place of residence (**B**); grade level (**C**); gender (**D**); following/not following a plant-based diet (**E**).

Figure 1 presents the level of respondents’ nutritional knowledge according to nutritional status (Figure 1A), place of residence (Figure 1B), grade level (Figure 1C), sex (Figure 1D), and adherence or non-adherence to a plant-based diet (Figure 1E). Statistically significant differences were observed only with respect to grade level, sex, and adherence to a plant-based diet. The analysis demonstrated that female students achieved significantly higher scores on the nutritional knowledge test compared with male students (*p* = 0.024). Moreover, significant differences in knowledge levels were identified between grade levels (*p* < 0.001). First-grade students obtained lower scores than students in the second, third, and fourth grades, whereas no significant differences were found among the latter three groups. Additionally, respondents who currently followed or had previously followed a plant-based diet achieved significantly higher scores on the nutritional knowledge test than students who had never adhered to such dietary patterns (*p* = 0.041).

The analysis of individual test items revealed specific areas of limited knowledge among students. The items with the lowest correct response rates concerned sources of non-haem iron absorption, vitamin B_12_ sources, the major sources of haem iron, vitamin B_12_ deficiency, the most difficult nutrients to balance in a plant-based diet, the fact that a vegetarian diet excludes animal fats, and sources of calcium in a plant-based diet, with correct responses ranging from 17.89% to 54.55% (Table 4). These findings indicate particular nutritional topics where adolescents’ understanding is relatively weak.

### 3.3. Frequency of Plant-Based Diets in the Study Population

The analyses revealed significant differences in the prevalence of plant-based diet adherence according to sex and level of nutritional knowledge. Adherence to a plant-based diet was reported significantly more frequently by female students than by male students (*p* = 0.006). Furthermore, respondents with a sufficient level of nutritional knowledge were more likely to follow plant-based diets compared with students demonstrating an insufficient knowledge (*p* = 0.017). No statistically significant associations were identified between adherence to plant-based diets and place of residence, body mass index, or grade level (Table 5).

### 3.4. Multivariate Analysis of Factors Associated with Sufficient Nutritional Knowledge

Multivariable logistic regression analysis demonstrated that grade level, sex, place of residence, and adherence to plant-based diets were significantly associated with a sufficient knowledge regarding plant-based diets. Compared with first-grade students, those in the third grade (aOR = 3.66; 95% CI: 1.52–8.81; *p* = 0.004) and fourth grade (aOR = 3.62; 95% CI: 1.39–9.39; *p* = 0.008) were significantly associated with higher odds of having a sufficient knowledge. No statistically significant differences were observed for second-grade students. Male sex was associated with significantly lower odds of attaining a sufficient knowledge compared with female sex (aOR = 0.38; 95% CI: 0.20–0.74; *p* = 0.004). Students residing in rural areas also had lower odds of achieving a sufficient knowledge compared with those living in large urban areas (aOR = 0.50; 95% CI: 0.26–0.94; *p* = 0.031). Furthermore, non-adherence to plant-based diets was associated with a significantly lower likelihood of possessing sufficient nutritional knowledge (aOR = 0.32; 95% CI: 0.11–0.95; *p* = 0.040). In contrast, body mass index (BMI) and residence in small towns were not significantly associated with the level of nutritional knowledge (Table 6).

## 4. Discussion

In the present study, both the knowledge regarding vegetarian and vegan diets and adherence to plant-based diets were assessed, along with their associations with selected socio-demographic and anthropometric characteristics. The majority of participants achieved a sufficient level of nutritional knowledge, with significant differences observed according to sex, grade level, and declared adherence to a plant-based diet.

Consistent with findings from other studies, female students demonstrated a higher knowledge regarding nutrition and plant-based diets than male students. Previous research has similarly indicated that females more frequently report greater interest in health and conscious dietary choices. In a Polish study conducted among primary school students, girls achieved higher scores on a nutritional knowledge test and higher values on the Pro-Healthy Diet Index, while simultaneously exhibiting lower values on the Unhealthy Diet Index [26]. Furthermore, Kołłajtis-Dołowy and Żamojcin (2016) reported that young men attained lower scores in nutritional knowledge compared with women, and more than 25% of them declared no intention to expand their knowledge regarding food and nutrition [27]. Differences in knowledge levels across grade levels may reflect the accumulation of educational experience and emotional maturation. Older students may be more interested in health-related topics and make more conscious dietary decisions, which aligns with observations in similar adolescent populations. Research conducted in Egypt indicated that nutritional knowledge increases with age during adolescence, with the highest scores observed among older adolescents (17–19 years), particularly in the domain of basic nutritional knowledge [28]. Similarly, Naeeni et al. (2014), comparing the nutritional knowledge of primary and middle school students, demonstrated a positive association between age and higher levels of nutritional knowledge among students in higher grades [29].

The findings regarding adherence to plant-based diets confirm that female students were significantly more likely to report following such dietary patterns, a trend that has also been observed in studies conducted among university students and young adults. Pfeiler and Egloff (2018) identified significant sex differences in the adoption of plant-based diets, with women declaring adherence to these diets significantly more often than men (*p* < 0.001) [30]. In contrast, men have been shown to consume meat more frequently and to demonstrate lower motivation to maintain plant-based dietary patterns [25,31].

The results of the present study indicate that ethical considerations were the primary motivation for adopting a plant-based diet among respondents, while environmental and health-related reasons were reported less frequently. This suggests that moral concerns may play a more prominent role than health-related factors in shaping dietary choices in this population. The literature emphasizes that motivations underlying the choice of plant-based diets are multidimensional, encompassing health-related, ethical, and environmental considerations, and often varying according to sex and age group. Compared with men, women more frequently express support for animal rights and are more likely to perceive meat consumption as having a negative environmental impact [32,33].

At the same time, a considerable proportion of participants reported short-term adherence to a plant-based diet (≤6 months), which may indicate an exploratory approach rather than long-term commitment. Importantly, only 20% of participants reported consulting a dietitian, while the majority had not sought professional guidance. This may raise concerns regarding the nutritional adequacy of self-managed plant-based diets, particularly among adolescents, and highlights the need for improved access to nutrition education and professional dietary support.

The association between the knowledge and adherence to plant-based diets is of particular interest. Our findings suggest that students with a higher level of nutritional knowledge were more likely to report following a plant-based diet, which may indicate that an understanding of nutrients and the principles of healthy eating facilitates the acceptance and implementation of such dietary patterns. The lowest scores were observed for items related to vitamin B_12_ sources, the major sources of haem iron, vitamin B_12_ deficiency, the most difficult to balance in a plant-based diet, the fact that a vegetarian diet excludes animal fats, and sources of calcium in a plant-based diet. This suggests that nutritional education programs for adolescents could benefit from targeted focus on these areas, emphasizing key nutrients that are critical in plant-based diets. Similar relationships have been observed in other studies, in which individuals adhering to plant-based diets demonstrated greater interest in health and nutritional principles compared with those following a traditional dietary pattern. Research involving respondents adhering to vegan, vegetarian, and semi-vegetarian diets has shown that individuals following plant-based dietary patterns exhibited both higher diet quality and greater nutritional knowledge (*p* < 0.001) [3]. However, due to the cross-sectional design of the present study, it is not possible to establish a causal relationship between nutritional knowledge and adherence to a plant-based diet.

The absence of a significant association between nutritional status (BMI) and knowledge is noteworthy, as some studies suggest that increased nutritional awareness may promote healthier body weight patterns. This finding may indicate that knowledge regarding plant-based diets does not necessarily translate into differences in BMI within the analyzed age group, which is consistent with literature emphasizing that environmental and behavioral factors also play a crucial role in shaping body weight. Similar findings were reported in studies conducted in China examining the relationship between nutritional knowledge and overweight and obesity among children and adolescents, where higher BMI was correlated with lower levels of nutritional knowledge [34]. In contrast, studies conducted in Poland identified a significant negative association between higher BMI and level of nutritional knowledge [24].

Furthermore, our findings regarding the frequency of adherence to plant-based diets according to place of residence—which revealed no significant differences—may reflect the increasing accessibility of information on plant-based diets through social media and broadly available health education among adolescents. Other studies suggest that young people, regardless of place of residence, have growing access to nutrition-related content, which may reduce disparities arising from local contextual factors. Nevertheless, some reports continue to indicate significant differences between students from rural areas and those residing in larger cities [29,35]. Although no significant differences were observed in the prevalence of plant-based diet adherence according to place of residence, multivariable analysis demonstrated that students living in rural areas had lower odds of achieving a sufficient level of nutritional knowledge compared with their peers from large urban centers. These findings may reflect disparities in access to structured nutrition education or health-promoting environments rather than differences in dietary choices per se.

The level of nutritional knowledge and dietary behaviors in adolescent populations constitutes an important determinant of future public health. As emphasized in epidemiological research, plant-based diets are increasingly chosen among children and adolescents and are associated with distinct dietary patterns that may influence both overall diet quality and the risk of nutrient deficiencies if not appropriately planned [2,4,36]. Compared with children following a traditional dietary pattern, those adhering to plant-based diets may face a higher risk of inadequate intake of certain vitamins and minerals. Vitamin B_12_ is of particular concern, as its blood concentrations have been shown to differ significantly between dietary groups (−97 pmol/L; 95% CI, −187 to −7; I^2^ = 98.5%), with the lowest levels observed among children following a vegan diet [37]. Although the present study did not assess the nutritional value of the participants’ diets, the available evidence underscores the importance of adequate nutritional knowledge and proper dietary planning among adolescents adhering to plant-based diets.

The findings should be interpreted as reflecting associations rather than causal relationships.

In the present study, the diversity of educational profiles within the examined school—ranging from humanities and psychology to natural sciences and multidisciplinary academic tracks—did not significantly differentiate the students in terms of their nutritional knowledge or dietary adherence. This lack of significant variation suggests that adolescent interest in and knowledge of plant-based diets may be driven more by extracurricular factors, such as personal health beliefs, ethical considerations, and the pervasive influence of social media, rather than formal school curricula alone. While students in science-oriented profiles might be expected to possess a deeper understanding of biological processes, the specific practicalities of planning a balanced vegetarian or vegan diet appear to be a subject of broader interest that transcends specific academic tracks. However, it should be noted that all participants attended a general secondary school, and future research should explore whether these patterns hold true in more technically or vocationally oriented educational settings, where health-related subjects may be emphasized differently. While our study did not find significant differences between educational tracks, other recent research among Polish secondary school students suggests that academic profile can be a strong predictor of nutritional literacy. For instance, a study conducted in the Tri-City area found that students in natural science tracks had significantly higher odds of correctly identifying the roles of dietary fiber and omega-3 fatty acids compared to their peers in other profiles [38]. This discrepancy may be due to regional differences or the specific focus of the knowledge assessment tools used in different studies.

### Limitations and Strengths of the Study

A notable limitation of this study is that it was conducted in a single general secondary school using a convenience sampling method, which may limit the generalizability of the findings to the broader adolescent population in Poland. This sampling approach introduces a potential selection bias, as the students who participated might have a greater pre-existing interest in nutrition than the general population. Students from other regions or different school types, such as technical or vocational schools, may present different levels of nutritional knowledge and dietary patterns. Furthermore, a significant limitation is the strong predominance of female participants (approx. 73%), which reflects the school’s demographics but may bias the results; research suggests that females typically possess higher nutritional awareness and are more likely to adopt plant-based diets [31].

A limitation of the study is its cross-sectional design, which does not permit conclusions regarding causal relationships. Additionally, data on dietary practices as well as body weight and height were based on self-reports rather than direct measurements. This may introduce reporting bias, particularly social desirability bias or the underestimation/overestimation of anthropometric values, which could affect the BMI-related analyses.

Moreover, the set of variables included in the analysis was limited to those available in the questionnaire and did not include potentially important omitted variables such as socioeconomic status, parental education, or family eating patterns. The absence of these factors means that residual confounding cannot be excluded, as home environment and parental health literacy are key drivers of adolescent dietary choices. Future research should include these socioeconomic predictors to provide a more holistic understanding.

Despite these limitations, a significant strength of this study is the inclusion of a relatively large and internally diverse sample of secondary school students, a population that remains underrepresented in plant-based diet research. By selecting a school with multiple educational tracks—including humanities, natural sciences, and psychology—we were able to capture a broad cross-section of academic interests. Moreover, the preliminary analysis confirming no significant differences between these profiles allowed for a robust, combined analysis of factors influencing nutritional knowledge, providing a solid foundation for future, larger-scale multi-center studies. The study simultaneously assessed both nutritional knowledge and adherence to vegetarian and vegan diets, providing a more comprehensive perspective on factors associated with adolescents’ dietary patterns. Moreover, the application of multivariable logistic regression enabled the identification of factors associated with sufficient nutritional knowledge, adjusted for variables included in the model of sufficient nutritional knowledge while controlling for potential confounding variables. The findings offer valuable insights into variables associated with nutritional knowledge and plant-based diet adherence among adolescents and may inform the development of targeted nutrition education and public health interventions.

## 5. Conclusions

Associations were observed between sufficient nutritional knowledge regarding vegetarian and vegan diets and several factors, including grade level, place of residence, and adherence to plant-based diets. Specifically, students in higher grades, adolescents residing in large urban areas, and individuals reporting adherence to plant-based diets tended to have higher knowledge scores. In addition, adherence to plant-based diets was associated with higher knowledge, highlighting the potential link between knowledge and dietary behaviors. However, causal relationships cannot be inferred due to the cross-sectional design of the study.

The analysis of item-level responses highlighted specific areas where knowledge was limited, particularly vitamin B_12_ sources, the major sources of haem iron, vitamin B_12_ deficiency, the most difficult nutrients to balance in a plant-based diet, the fact that a vegetarian diet excludes animal fats, and sources of calcium in a plant-based diet. These findings suggest that targeted educational programs addressing these nutrient areas could support adolescents’ understanding of plant-based nutrition and promote informed dietary choices.

The study provides insights into patterns of nutritional knowledge among secondary school students, which may inform the development of tailored interventions aimed at improving awareness and competence in planning balanced plant-based diets.

## Figures and Tables

**Table 1:** Socio-demographic characteristics of the study group.

Variable	N = 341	[%]
Sex		
Woman	249	73.02
Man	80	23.46
Does not want to specify	12	3.52
Place of residence		
Village	84	24.64
Small town (<500,000 inhabitants)	35	10.26
Big city (≥500,000 inhabitants)	222	65.10
BMI		
Below normal weight	21	6.16
Normal weight	284	83.28
Above normal weight	36	10.56
Grade level		
1	64	18.77
2	100	29.32
3	101	29.62
4	76	22.29

**Table 2:** Characteristics of students adhering to plant-based diets (N = 55).

Parameter	Category	N = 55	[%]
Duration of diet	<6 months	18	32.73
	6–12 months	11	20.00
	1–2 years	11	20.00
	>2 years	15	27.27
Primary Motivation *	Ethical	40	72.73
	Environmental	16	29.09
	Health	8	14.54
Consulted a Dietitian	Yes	11	20.00
	No/Planning to	44	80.00

* multiple-choice question

**Table 3:** Knowledge test results and nutritional knowledge classification.

Variable	N	%	Average ± SD	Median (Q_1_; Q_3_)	Min–Max
Knowledge test result (points)	341	100	17.92 ± 2.78	18 (16; 20)	9–25
Knowledge			-	-	-
Insufficient	64	18.77			
Sufficient	277	81.23			

**Table 4:** Items with the lowest correct response rates in the plant-based diet knowledge test.

Item No.	Question (Short Version)	% Correct
1	Non-haem iron absorption	17.89
2	Vitamin B_12_ sources	26.69
3	The major sources of haem iron	45.75
4	Vitamin B_12_ deficiency	49.27
5	The most difficult to balance in a plant-based diet	50.15
6	A vegetarian diet—excludes animal fats	54.25
7	Sources of calcium in a plant-based diet	54.55

**Table 5:** Frequency of plant-based diets depending on selected socio-demographic characteristics and level of nutritional knowledge (N = 341).

Variable	I Follow a Plant-Based Diet N [%]	I Don’t Follow a Plant-Based Diet N [%]	*p*
Place of residence			0.701
Village	12 (3.52)	72 (21.11)	
Small town	7 (2.05)	27 (7.92)	
Big city	36 (10.56)	187 (54.84)	
Sex			0.006
Woman	48 (14.08)	202 (59.24)	
Man	4 (1.17)	76 (22.29)	
BMI			0.170
Below normal weight	6 (1.76)	14 (4.11)	
Normal weight	45 (13.20)	240 (70.38)	
Above normal weight	4 (1.17)	32 (9.38)	
Grade level			0.371
1	15 (4.40)	49 (14.37)	
2	14 (4.11)	86 (25.22)	
3	15 (4.40)	86 (25.22)	
4	11 (3.23)	65 (19.06)	
Knowledge			0.017
Sufficient	51 (14.96)	226 (66.28)	
Insufficient	4 (1.17)	60 (17.60)	

The category ‘I follow a plant-based diet’ includes both current and former practitioners (ever-adhered) of vegetarian or vegan diets. The category ‘I don’t follow a plant-based diet’ refers to participants who have never adhered to such dietary patterns.

**Table 6:** Multivariate logistic regression analysis of factors associated with sufficient knowledge about plant-based diets.

Variable	*p*	aOR	95% CI	
			Lower Limit	Upper Limit
BMI < N	0.566	1.573	0.335	7.393
BMI > N	0.980	1.012	0.413	2.475
G2	0.417	1.374	0.638	2.956
G3	0.004	3.656	1.517	8.811
G4	0.008	3.617	1.393	9.389
M	0.004	0.383	0.198	0.740
V	0.031	0.496	0.262	0.939
ST	0.811	0.885	0.326	2.403
DF	0.040	0.321	0.108	0.951

Reference categories: Sex = W (Woman); Grade level = Grade 1; BMI = Normal body weight; Place of residence = Big city (≥500,000 inhabitants); Plant-based diet = I follow a plant-based diet; BMI < N—BMI below normal; BMI > N—BMI above normal; G2—Class 2; G3—Class 3; G4—Class 4, M—Man; V—Village; ST—Small town; DF—I don’t follow a plant-based diet.

## Data Availability

The original contributions presented in this study are included in the article. Further inquiries can be directed to the corresponding author.

## Supplementary Materials

The following supporting information can be downloaded at: https://www.mdpi.com/article/10.3390/nu18081210/s1: S1: Questionnaire.
