# Supplementary material for: Conditions for Knowledge and Application of Vegetarian/Vegan Diets Among Secondary School Students: A Cross-Sectional Study
Source: Nutrients. 2026 Apr 11;18(8):1210. doi: 10.3390/nu18081210 (PMC13118344; doi:10.3390/nu18081210)
Supplement: Supplementary file 1 [file nutrients-18-01210-s001.zip › nutrients-4185058-supplementary.pdf]

Supplementary Material: S1 - Questionnaire

I. PERSONAL DETAILS

1. How old are you?

   …………………………..

2. How tall are you?

   …………………………..

3. What is your weight?

   …………………………..

4. Which grade are you currently in?

   …………………………..

5. What is the profile of your class?

   …………………………..

6. What is your gender?
   - Female
   - Male
   - Prefer not to say

7. Place of residence.
   - Village
   - Town with up to 10,000 inhabitants
   - Town with 10,000–100,000 inhabitants
   - Town with 100,000–500,000 inhabitants
   - Town with over 500,000 inhabitants

II. NUTRITIONAL KNOWLEDGE

1. The way of eating on a vegetarian and vegan diet is the same. (*Only one answer can be selected)*
   - True
   - False
   - I don't know

2. A vegetarian diet excludes the consumption of: *(More than one answer can be selected)*
   - Meat and meat products
   - Eggs
   - Dairy products (e.g., milk, yogurt, cream, cheese)
   - Cereal products (e.g., groats, pasta, bread, rice)

- Fish and seafood
- Vegetables
- Fruits
- Legumes (e.g., beans, broad beans, peas, lentils, chickpeas, soybeans)
- Plant-based fats (e.g., rapeseed oil, sunflower oil, olive oil, margarine)
- Animal fats (e.g., lard, butter)
- Sweets
- Nuts and seeds

3. A vegan diet excludes the consumption of: *(More than one answer can be selected)*
   - Meat and meat products
   - Eggs
   - Dairy products (e.g., milk, yogurt, cream, cheese)
   - Cereal products (e.g., groats, pasta, bread, rice)
   - Fish and seafood
   - Vegetables
   - Fruits
   - Legumes (e.g., beans, broad beans, peas, lentils, chickpeas, soybeans)
   - Plant-based fats (e.g., rapeseed oil, sunflower oil, olive oil, margarine)
   - Animal fats (e.g., lard, butter)
   - Sweets
   - Nuts and seeds

4. The main source of calcium in a vegetarian/vegan diet is: *(Only one answer can be selected)*
   - Poppy seeds, almonds, soy
   - Carrot, banana, walnuts
   - Soy, tomato, broccoli

5. Haem iron is found in the highest amount in: *(Only one answer can be selected)*
   - Beef
   - Beans
   - Spinach
   - Walnuts

6. Non-haem iron is found in the highest amounts in: *(Only one answer can be selected)*
   - Milk
   - Soya
   - Rice
   - Tomato

7. Which of the following nutrients increases the absorption of non-haem iron? *(Only one answer can be selected)*
   - Magnesium
   - Vitamin C

- Phosphorus
- Vitamin D

8. Vitamin B_{12} is found only in animal-based products. *(Only one answer can be selected)*
   - True
   - False
   - I don't know

9. The main source of complete protein in a vegetarian/vegan diet is: *(Only one answer can be selected)*
   - Soya
   - Buckwheat groats
   - Nuts
   - Oat Flakes

10. Vitamin D increases calcium absorption. *(Only one answer can be selected)*
    - True
    - False
    - I don't know

11. The main source of omega-3 fatty acids in a vegetarian/vegan diet is: *(Only one answer can be selected)*
    - Oily fish (e.g. salmon, trout)
    - Vegetable oils (e.g. linseed oil, rapeseed oil)
    - Butter and margarine
    - Cereal products (e.g. porridge, pasta)

12. In a vegetarian/vegan diet, meat should be replaced with other foods to ensure an adequate intake of nutrients. *(Only one answer can be selected)*
    - True
    - False
    - I don't know

13. Following a vegetarian/vegan diet may have a negative impact on human health. *(Only one answer can be selected)*
    - True, always
    - True, if the diet is poorly balanced
    - False
    - I don’t know

14. A vegetarian/vegan diet can be a nutritionally complete diet (i.e. providing all nutrients in the right amounts). *(Only one answer can be selected)*
    - True, always
    - True, if the diet is poorly balanced
    - False
    - I don’t know

15. A vegetarian/vegan diet requires supplementation. (*Only one answer can be selected)*
    - True, always
    - True, if the diet is poorly balanced
    - False
    - I don’t know

16. Which of the following nutrients is essential for the health of bones and teeth? (*Only one answer can be selected)*
    - Iron
    - Vitamin C
    - Calcium
    - Magnesium

17. Vitamin D is fat-soluble. *(Only one answer can be selected)*
    - True
    - False
    - I don't know

18. A vitamin B_{12} deficiency most commonly leads to: *(Only one answer can be selected)*
    - Scurvy
    - Osteoporosis
    - Megaloblastic anaemia
    - Rickets

19. What is the most difficult aspect of a vegetarian/vegan diet to balance? *(Only one answer can be selected)*
    - Vitamin B_{12}, iron, calcium
    - Vitamin C, magnesium, protein
    - Vitamin B_{12}, protein, dietary fiber

20. Where do you get your information about vegetarian/vegan diets? *(More than one answer can be selected)*
    - The internet (websites, blogs, forums)
    - Social media (Instagram, TikTok, YouTube)
    - Teachers at school
    - Family and friends
    - Books and academic articles
    - Other (please specify)..

21. Do you currently follow, or have you ever followed, a vegetarian or vegan diet? *(Only one answer can be selected)*
    - Yes, I follow it.
    - Yes, I used to follow it.
    - No

If you answered ‘Yes, I follow’ or ‘Yes, I used to follow’ to question 21, please move on to Part III of the questionnaire.

III. QUESTIONS FOR PEOPLE WHO FOLLOW OR HAVE FOLLOWED A VEGETARIAN OR VEGAN DIET.

1. Which type of vegetarian diet do you follow/have you followed? *(Only one answer can be selected)*
    - Lacto-ovo-vegetarianism (exclusion of meat, fish and seafood; dairy products, honey and eggs are permitted)
    - Lacto-vegetarianism (exclusion of meat, fish, seafood, honey and eggs; dairy products are permitted)
    - Ovo-vegetarianism (exclusion of meat, fish, seafood, honey and dairy products; eggs are permitted)
    - Veganism (exclusion of meat, fish, seafood, dairy products, honey and eggs)
    - Raw foodism (this diet consists exclusively of raw plant-based foods)
    - Fruitarianism (exclusion of meat, fish, seafood, dairy, honey, eggs and products obtained as a result of the ‘death’ (destruction) of a plant)

2. How long have you been following your diet? *(Only one answer can be selected)*
    - Less than 6 months
    - 6–12 months
    - 1–2 years
    - More than 2 years

3. What made you choose this diet? *(More than one answer can be selected)*
    - Health concerns
    - Ethical concerns
    - Environmental concerns
    - Other (please specify)..

4. How often do you eat foods rich in plant-based protein (e.g. legumes, tofu, tempeh)? *(Only one answer can be selected)*
    - Every day
    - Several times a week
    - Several times a month

- Several times a year
- Never

5. How often do you eat foods fortified with vitamin B12? *(Only one answer can be selected)*
   - Every day
   - Several times a week
   - Several times a month
   - Several times a year
   - Never

6. How often do you eat foods rich in plant-based iron (e.g. legumes, pumpkin seeds, quinoa)? *(Only one answer can be selected)*
   - Every day
   - Several times a week
   - Several times a month
   - Several times a year
   - Never

7. How often do you eat plant-based foods rich in calcium (e.g. plant-based drinks, nuts, tofu, green vegetables)? *(Only one answer can be selected)*
   - Every day
   - Several times a week
   - Several times a month
   - Several times a year
   - Never

8. Have you discussed your diet with a dietitian or doctor? *(Only one answer can be selected)*
   - Yes
   - No
   - I'm planning to do it

9. Do you know your daily energy requirements? *(Only one answer can be selected)*
   - Yes
   - No

10. Do you regularly check your vitamin and mineral levels (e.g. through blood or urine tests)? *(Only one answer can be selected)*
    - Yes
    - No

11. Have you noticed any weight loss whilst following a vegetarian or vegan diet? *(Only one answer can be selected)*
    - Yes

- No

12. Have you experienced any health issues that might be related to your diet (e.g. fatigue, weakness, headaches, concentration problems)? *(Only one answer can be selected)*
    - Yes
    - No
    - I'm not sure

13. If so, which of the following health problems have you experienced? *(More than one answer can be selected)*
    - Fatigue
    - Headaches
    - Weakness
    - Concentration problems
    - Skin problems
    - Other (please specify)..

14. Are you taking any medication or supplements (e.g. vitamin B12, vitamin D, omega-3 fatty acids)? *(Only one answer can be selected)*
    - Yes
    - No

15. If so, how often? *(Only one answer can be selected)*
    - Every day
    - Several times a week
    - Several times a month
    - Several times a year
    - Never

ANSWERS FOR PART II:

QUESTION NUMBER	ANSWER
1	B
2	A, E, J
3	A, B, C, E, J
4	A
5	A
6	B
7	B
8	A
9	A
10	A
11	B
12	A
13	B
14	B
15	B
16	C
17	A
18	C
19	A
